# Artemisinin May Disrupt Hyphae Formation by Suppressing Biofilm-Related Genes of *Candida albicans*: In Vitro and In Silico Approaches

**DOI:** 10.3390/antibiotics13040310

**Published:** 2024-03-28

**Authors:** Esra Sumlu, Merve Aydin, Emine Nedime Korucu, Saliha Alyar, Ahmed Moustapha Nsangou

**Affiliations:** 1 Department of Medical Pharmacology, Faculty of Medicine, KTO Karatay University, 42020 Konya, Turkey; esra.sumlu@karatay.edu.tr; 2 Department of Medical Microbiology, Faculty of Medicine, KTO Karatay University, 42020 Konya, Turkey; 3 Department of Molecular Biology and Genetics, Faculty of Science, Necmettin Erbakan University, 42090 Konya, Turkey; enkorucu@erbakan.edu.tr; 4 Department of Chemistry, Faculty of Science, Karatekin University, 18100 Çankırı, Turkey; saliha@karatekin.edu.tr; 5 Department of Medical Microbiology, Faculty of Medicine, Selçuk University, 42130 Konya, Turkey; lemoust5@gmail.com

**Keywords:** artemisinin, *Candida albicans*, biofilm-related genes, molecular docking, FESEM

## Abstract

This study aimed to assess the antifungal and antibiofilm efficacy of artemisinin against *Candida (C.)* species, analyze its impact on gene expression levels within *C. albicans* biofilms, and investigate the molecular interactions through molecular docking. The antifungal efficacy of artemisinin on a variety of *Candida* species, including fluconazole-resistant and -susceptible species, was evaluated by the microdilution method. The effect of artemisinin on *C. albicans* biofilm formation was investigated by MTT and FESEM. The mRNA expression of the genes related to biofilm was analyzed by qRT-PCR. In addition, molecular docking analysis was used to understand the interaction between artemisinin and *C. albicans* at the molecular level with RAS1-cAMP-EFG1 and EFG1-regulated genes. Artemisinin showed higher sensitivity against non-albicans *Candida* strains. Furthermore, artemisinin was strongly inhibitory against *C. albicans* biofilms at 640 µg/mL. Artemisinin downregulated adhesion-related genes ALS3, HWP1, and ECE1, hyphal development genes UME6 and HGC1, and hyphal CAMP-dependent protein kinase regulators CYR1, RAS1, and EFG1. Furthermore, molecular docking analysis revealed that artemisinin and EFG1 had the highest affinity, followed by UME6. FESEM analysis showed that the fluconazole- and artemisinin-treated groups exhibited a reduced hyphal network, unusual surface bulges, and the formation of pores on the cell surfaces. Our study suggests that artemisinin may have antifungal potential and showed a remarkable antibiofilm activity by significantly suppressing adhesion and hyphal development through interaction with key proteins involved in biofilm formation, such as EFG1.

## 1. Introduction

*Candida (C.)* species are commensal fungi that are resident in the skin, mouth, gastrointestinal tract, and vaginal microbiota of healthy humans [1]. However, these species can cause severe invasive mucosal and systemic infections with high mortality rates in immunocompromised hosts [1,2]. Although *C. albicans* is the most common cause of superficial and severe systemic fungal infections, non-albicans *Candida* species such as *C. krusei*, *C. glabrata,* and *C. parapsilosis* have also emerged as significant contributors [2,3]. 

The biofilm-forming ability of *C. albicans* is one of the key factors contributing to its virulence. *Candida albicans* can develop biofilms on mucosal surfaces and implanted medical devices, which can result in the progression of clinical infections to invasive systemic infections [4,5]. *Candida* biofilms are intricate communities composed of a variety of cell types (yeast-like, pseudohyphal, and hyphal cells) and the protective extracellular matrix. This multi-element structure of the biofilm causes an increase in antifungal drug concentration and resistance [5,6,7]. Moreover, the presence of the extracellular matrix not only protects the cells within the biofilm but also acts as a physical barrier against immune cells and antifungal agents [6,7]. Therefore, biofilm-associated infections often require more aggressive and long-term treatment strategies than their planktonic counterparts. However, at the high doses required for antibiofilm activity, almost all antifungals can also be hepatotoxic and nephrotoxic [7,8]. 

The yeast-to-hyphae dimorphism of *C. albicans* is another virulence factor associated with biofilm formation and cell adhesion. Hyphal development is critical for biofilm formation and immune evasion, as it promotes colonization, adhesion, cell penetration, and phagocytosis resistance [9]. 

*Candida albicans* infections are commonly treated with three primary antifungal classes: azoles, polyenes, and echinocandins. In the azoles, fluconazole (FLC) is commonly used for prophylactic and therapeutic purposes owing to its favorable properties such as high efficacy, bioavailability, low toxicity, and affordability [7]. However, the widespread and frequent administration of FLC in clinical settings reduces its efficacy by promoting the development of FLC-resistant strains [10,11]. This resistance can be attributed to several mechanisms, including mutations in target enzymes, transcription factors or transporters, and enhanced efflux pump activity [11]. Moreover, *Candida* biofilm is resistant to most of these standard antifungals, and only a few molecules that effectively target the biofilm have been identified. New antifungal and antibiofilm agents are needed to address the emerging problem of increasing resistance and severe infections [12]. High costs have impeded the development of potential therapeutics in recent years. As a result, drugs that are already on the market have become an important alternative in terms of time and cost in the search for new treatment options. It also provides a safe toxicity profile for these drugs [12,13]. 

Given the challenges of drug discovery and treatment failure rates, repurposing traditional Chinese herbal medicines to treat various diseases is a promising strategy [14]. Artemisinin is a sesquiterpene lactone derived from the traditional Chinese herb *Artemisia annua* L. Due to its high efficacy and tolerability, artemisinin is included in first-line antimalarial regimens [15]. Aside from antimalarial activity, artemisinin and its derivatives have been reported to have antibacterial, antiviral, anti-inflammatory, and antitumor properties [16]. Several studies evaluated the antifungal effect of artemisinin and its derivatives on *Candida* spp. [17,18,19,20,21,22,23,24,25]. However, the exact mechanism underlying this antifungal and antibiofilm effect of artemisinin remains unknown. This study investigated the potential antifungal and antibiofilm effects of artemisinin with molecular aspects. We aimed to elucidate the therapeutic potential of artemisinin by investigating its activity against a broad variety of fluconazole-resistant and fluconazole-sensitive *Candida* species, as well as its effect on gene expression levels and interactions in *C. albicans* biofilms by molecular docking. Furthermore, this approach to the off-label use of artemisinin may offer new avenues for antifungal and antibiofilm therapy.

## 2. Results

### 2.1. Artemisinin Exhibits Antifungal Activity on Candida *spp.*

To assess the antifungal activity of artemisinin on *Candida* species, we determined the minimum inhibitory concentration (MIC) values of the compound using a collection of *Candida* strains that included fluconazole-susceptible, fluconazole-susceptible dose-dependent (SDD), and fluconazole-resistant isolates. The MIC values of artemisinin and fluconazole for three reference strains and forty-three clinical isolates of *Candida* spp. are presented in Table S1. Our collection included six fluconazole-resistant and six fluconazole-SDD strains. *C. krusei* and *C. guilliermondii* strains were more susceptible to artemisinin with MIC values in the range of 5−10 µg/mL and 20 µg/mL, respectively. The MIC values for *C. kefyr* isolates were found to be 80 µg/mL, while *C. tropicalis* and *C. lusitaniae* isolates displayed higher MIC values, ranging from 160 to 320 µg/mL and 160 to 640 µg/mL, respectively. For *C. albicans* strains, artemisinin showed a broad spectrum of antifungal activity with MIC values ranging from 160 to 1280 µg/mL. Notably, artemisinin displayed comparable antifungal activity against fluconazole-SDD and -resistant strains, similar to its activity against fluconazole-sensitive *Candida* strains (Table 1).

### 2.2. Artemisinin Inhibits Biofilm Formation of C. albicans

The biofilm production ability of 28 *C. albicans* strains was tested using the crystal violet staining method. A total of 8 (28.6%) of the 28 *C. albicans* strains were classified as strong biofilm producers (n = 8, seven clinical strains and *C. albicans* ATCC 10231).

In the present study, the antibiofilm potential of artemisinin was appraised. For this purpose, eight strong biofilm producers *of C. albicans* strains were exposed to 0.5–1280 concentrations of artemisinin for 24 h. The biofilm’s metabolic activity was analyzed using the MTT assay. Biofilms were inhibited by 91.2% when treated with 2560 µg/mL artemisinin. Approximately 78.5%, 50%, and 40.2% biofilm inhibition effects were obtained at artemisinin concentrations of 1280, 640, and 320 µg/mL, respectively. The MTT assay revealed that artemisinin effectively eliminated biofilms allowed to grow for 24 h and that the effect was dose-dependent. Hence, 640 μg/mL was considered the minimal biofilm inhibitory concentration of 50% (MBIC50) of artemisinin against *C. albicans*. The MBIC50 value of FLC was determined as 4 µg/mL.

### 2.3. Artemisinin Affects Hyphae, Adhesion, and Biofilm-Related Gene Expression in C. albicans 

**Figure 1:**
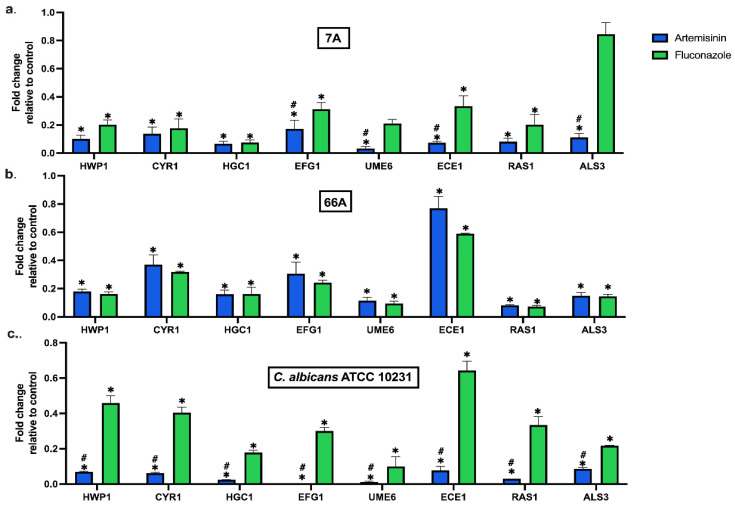
Effect of artemisinin on the expression of biofilm-related genes in strain (**a**) 7A, (**b**) 66A, and (**c**) *C. albicans* ATCC 10231. 18S ribosomal RNA was used for normalization of gene expression levels. Values are expressed as mean ± SEM, * *p* < 0.05, significantly different from the control; # *p* < 0.05, significantly different from the fluconazole.

Artemisinin significantly altered the expression of hypha, adhesion, and biofilm-related genes at MBIC50 (Figure 1). Artemisinin markedly reduced the expression of adhesion-related genes ALS3, HWP1, and ECE1 in all strains compared to the control. In addition, the hyphal development genes UME6 and HGC1 were significantly downregulated compared to the control. Moreover, artemisinin downregulated the hyphal CAMP-dependent protein kinase pathway regulators CYR1, RAS1, and EFG1, which are important pathways in forming *C. albicans* biofilms. Notably, EFG1 expression in the standard strain was nearly eliminated by artemisinin treatment. 

In clinical strains, the inhibitory effects of artemisinin and FLC on genes were similar. However, artemisinin significantly reduced the mRNA expression of EFG1, UME6, and ALS3 in 7A compared to FLC. The effect of artemisinin on gene expressions was significantly downregulated in *C. albicans* ATCC 10231 compared to FLC. Overall, artemisinin significantly affected the expression of genes linked to biofilm development in *C. albicans*. These results underline the potential of artemisinin as a therapeutic approach for treating infections associated with *C. albicans* biofilms.

### 2.4. Molecular Docking Studies

**Figure 2:**
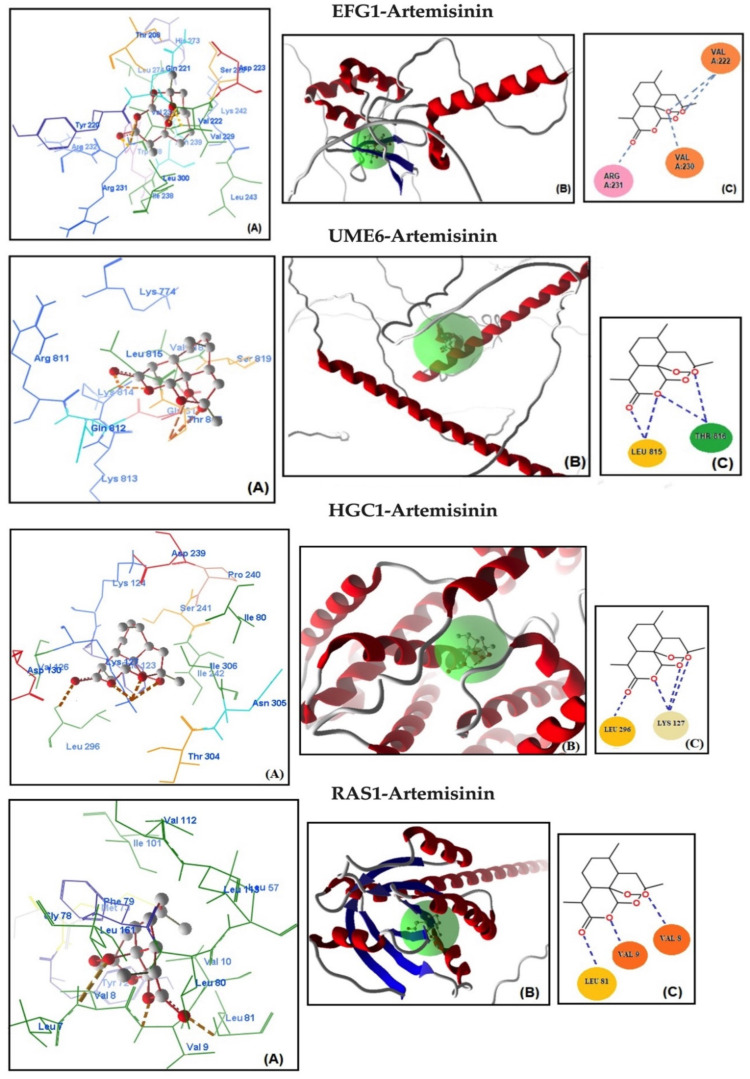

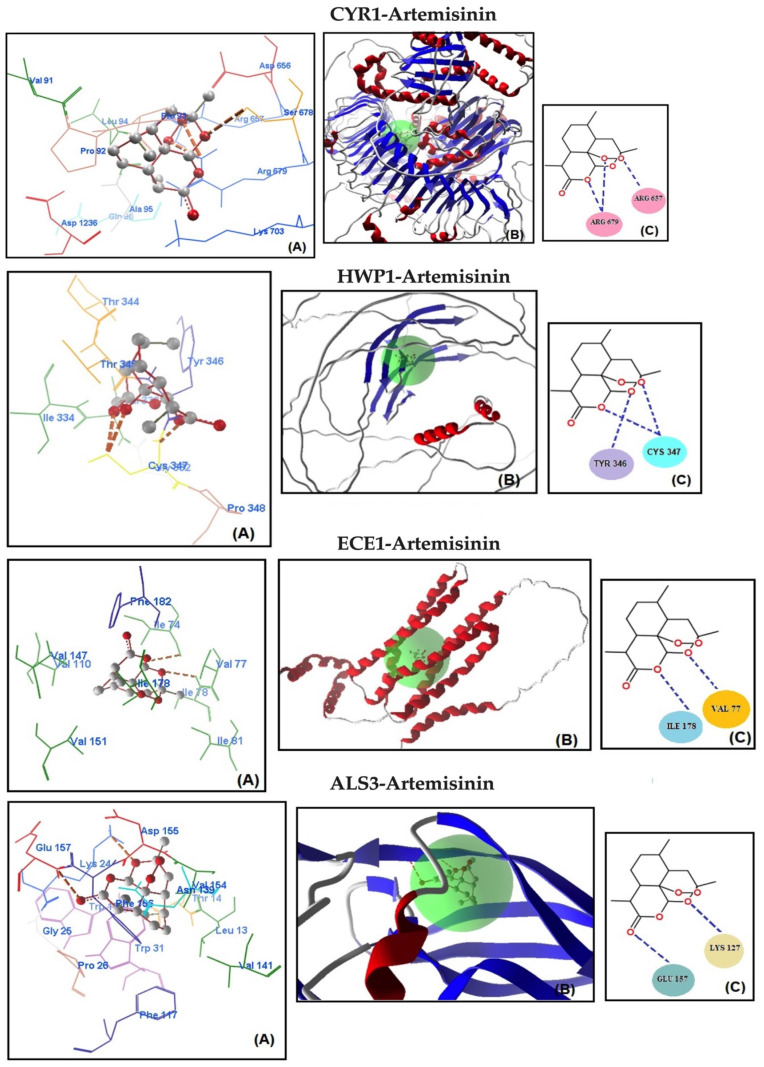
Molecular association of artemisinin with EFG1, UME6, HGC1, RAS1, CYR1, HWP1, ECE1, and ALS3 in *C. albicans* ATCC 10231. Each graph shows (**A**) molecular binding mode (dashed thick orange line, hydrogen bonds; unbound amino acids, steric interactions); (**B**) 3D interaction docked pose view (green area: active site); (**C**) two-dimensional representation of hydrogen bonds made by artemisinin and related protein.

Molecular docking analysis was conducted to assess the potential binding affinity of artemisinin to selected biofilm-related factors of *C. albicans*. The selected receptors were RAS1-cAMP-EFG1 genes (RAS1, CYR1, and EFG1) and EFG1-regulated genes (ALS3, HWP1, ECE1, UME6, and HGC1; Table 2). The total energies of the artemisinin–gene pairs ranged from −128.187 to −110.144 kcal/mol, while their full compatibility MolDock scores ranged from −129.466 to −113.367. The results showed that artemisinin–EFG1 had the highest affinity with a MolDock score of −129.466. This was followed by UME6 (total energy; −127.199 kcal/mol) with a MolDock score of −126.667 and HGC1 (total energy; −125.250 kcal/mol) with a MolDock score of −122.299 (Figure 2 and Table 2). Four hydrogen bonds were formed between artemisinin and the EFG1 gene. The first is between the C=O oxygen of artemisinin and the NH proton of amino acid Arg 231. The second is between the C-O-O oxygen of artemisinin and the NH proton of amino acid Val 222. The third hydrogen bond is between the C-O-O oxygen of artemisinin and the NH proton of the amino acid Val 222. The fourth hydrogen bond is between the C-O-O oxygen of artemisinin and the NH proton of the amino acid Val 230. The interactions of artemisinin with other genes are shown in Table 2.

**Figure 3:**
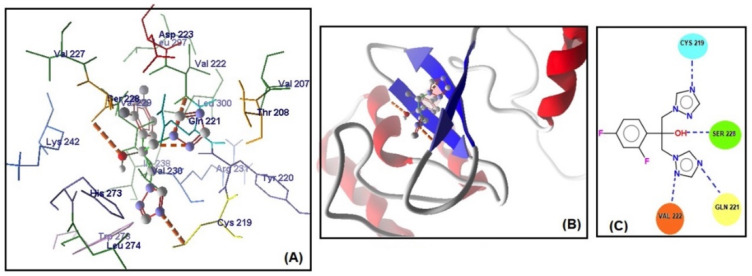
(**A**) Molecular binding mode of EFG1 and FLC (dashed thick orange line, hydrogen bonds; unbound amino acids, steric interactions); (**B**) the 3D interaction docked pose view (green area: active site); (**C**) two-dimensional representation of hydrogen bonds made by FLC and EFG1.

The MolDock scores indicate a concordance between the docking outcomes and the experimental findings. Furthermore, the docking analyses suggest that steric interactions and hydrogen bonding are crucial for the interaction between artemisinin and the active site of the target gene. Molecular docking studies of the most active gene, EFG1, were performed with artemisinin and the standard drug FLC (Figure 3). The docking study observed that the EFG1 gene exhibited similar binding affinity with both artemisinin and FLC (total energy: −128.199 kcal/mol; MolDock score: −129.786, ΔGbind: −9,21 kcal/mol and four hydrogen bonds). These results suggest that artemisinin can potentially target these receptors and may be useful as an antifungal agent.

### 2.5. Molecular Dynamic Simulation

**Figure 4:**
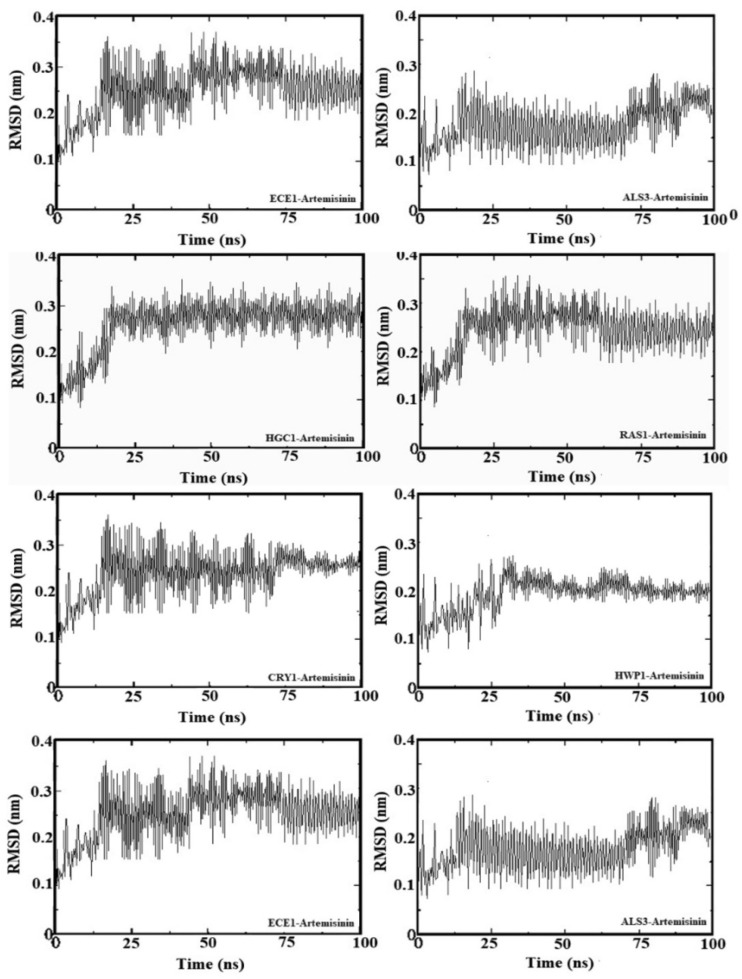
Root mean square deviation (RMSD) values of genes–artemisinin.

To further analyze the binding behavior of artemisinin to selected biofilm-related factors of *C. albicans*, molecular dynamics was utilized with GROMACS 2020 software. The selected receptors (RAS1, CYR1, EFG1, ALS3, HWP1, ECE1, UME6, and HGC1) were subjected to 100 ns of MDS. Figure 4 shows the root mean square deviation (RMSD) in Å and the equilibration of the systems during the 20 ns of MDS. The radius of gyration analysis reveals an upward pattern between 0 ns and 20 ns and then stable values until the end of the simulation. Overall, this demonstrates small fluctuations (less than 1 Å) during the simulation. This shows the compactness and stability of the receptor–ligand system. These results suggest that artemisinin can potentially target these receptors and be considered a potential antifungal agent, as in molecular docking studies. We used GROMACS 20 to calculate the binding free energy for the association of inhibitors. The calculated binding free energies are shown in Table 2. The binding free energy of the artemisinin–gene pairs ranged from −9.02 to −6.86 kcal/mol. When evaluating binding free energies, they appear compatible with experimental and molecular docking results. Artemisinin–EFG1 was found to have the highest affinity with a binding free energy of −9.02 kcal/mol.

### 2.6. Artemisinin Impaired Cytopathological Damage to C. albicans Biofilms

**Figure 5:**
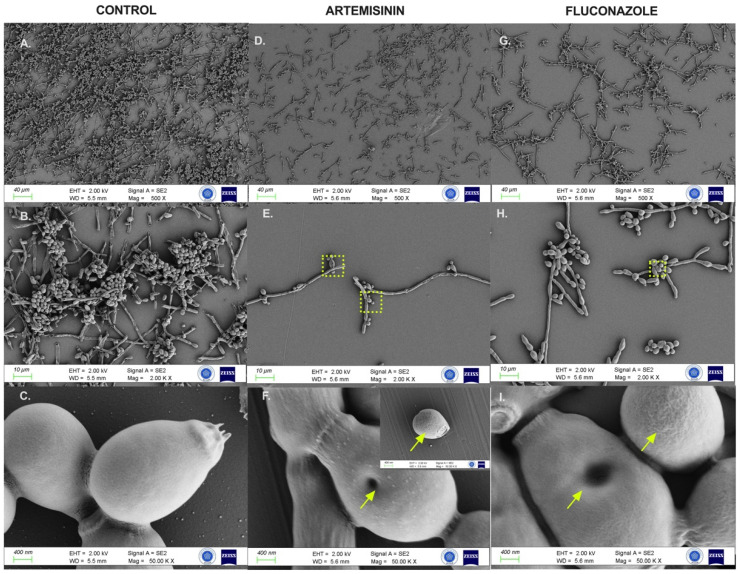
FESEM images of *C. albicans* ATCC 10231 biofilms after control and 24 h exposure to artemisinin and fluconazole. (**A**–**C**)**.** Untreated biofilm culture. (**D**–**F**)**.** Biofilm culture treated with 640 μg/mL artemisinin. (**G**–**I**)**.** Biofilm culture treated with 4 μg/mL fluconazole. Magnification scales of 500×, 2000×, and 50,000× were used for imaging. Arrows indicate unusual surfaces and pore formation. The frame shows the selected region.

FESEM analysis assessed the morphological and ultrastructural changes in *C. albicans* cells before and after treatment with artemisinin and FLC (Figure 5). FESEM images revealed significant differences in fungal morphology between untreated (control) and artemisinin- and FLC-treated cultures. In the control group, *C. albicans* showed a compact hyphal cell network. Intact *C. albicans* exhibited a typical cylindrical-shaped cell state with a smooth membrane surface. In contrast, the artemisinin group showed a reduced hyphal network, and *C. albicans* lost cell surface smoothness and showed unusual surface bulges and pore formation on cell surfaces, similar to the fluconazole group.

## 3. Discussion

The mechanism of antimalarial activity of artemisinin has not been definitively elucidated, but the strongest suggestion is based on the breakdown of endoperoxide in artemisinin, which causes cellular ROS accumulation and mitochondrial damage [26]. Artemisinin has also been reported to deplete cellular iron and form an iron–artemisinin complex within the parasite [27,28]. Iron acquisition mechanisms are vital in *C. albicans*, as in parasites [29]. Iron is also associated with the virulence of *C. albicans*, such as colonization and hyphae formation [30]. Iron limitation or iron chelators have increased the in vitro activity of various antifungal agents in *C. albicans* [31]. Consistently, artemisinin and its derivatives have been shown to increase susceptibility to antifungal drugs such as amphotericin, fluconazole, and miconazole [18,23]. In addition, several studies have reported on the antifungal effect of artemisinin with inconsistent results. Most of these studies have focused on standard strains of *C. albicans* or a few clinical samples, with limited interest in other *Candida* species [18,19,24].

A limited number of studies also report the antibiofilm properties of artemisinin [25]. Among all studies, only a limited number of genes have been examined for the antibiofilm effect of artemisinin or derivatives. Our study revealed that artemisinin had an antifungal effect on three standard and forty-three clinical *Candida* strains at high doses, but artemisinin showed a remarkable antibiofilm activity by significantly suppressing genes related to adhesion and hyphal exchange. To determine the activity of artemisinin on biofilm-associated structures, our results were supported by molecular docking studies and confirmed microscopically by the FESEM study.

Considering the studies on the antifungal effects of artemisinin, an early study reported that artemisinin has no antifungal activity [17]. However, some studies have shown that artemisinin has antifungal activity at concentrations as high as 100 µM [22]. Zhu et al. demonstrated that the MIC50 of artemisinin was above 200 mg/L in wild-type strains and 75 clinical *C. albicans* isolates [23]. Similarly, a recent study reported that the growth inhibitory activity of seven artemisinin derivatives, including artemisinin, was higher than 200 µg/mL in standard and clinical *C. albicans* strains, including azole-resistant strains [25]. However, the exact MIC value of artemisinin was not given in either study. Consistent with these previous studies, our study revealed that artemisinin showed antifungal activity against *C. albicans* strains at varying concentrations ranging from 160 to 1280 µg/mL. Artemisinin showed a remarkably high and wide range of antifungal activity, which may indicate potential fungistatic rather than fungicidal activity of artemisinin, as reported by Galal et al. [17]. In contrast, Das et al. reported that the MIC90 value of artemisinin in clinical *Candida* species was considerably lower (21.83–142.1 µg/mL) compared to these studies [19]. The variability of artemisinin MIC concentrations may be related to the clinical nature of the strains, passage-induced changes, or purity of the active substance. However, there are conflicting findings in the literature regarding the antifungal activity of artemisinin.

To date, there are a limited number of studies investigating the antifungal activity of artemisinin against non-albicans *Candida* species. Zhu et al. identified the antifungal properties of artemisinin and its derivatives through a high-throughput screening of the FDA-approved drug library. The researchers also showed that the artemisinin derivative artesunate inhibits *C. glabrata* growth. Furthermore, they created drug-resistant *C. glabrata* mutants to investigate the mechanism of action of artemisinin on *C. glabrata*, revealing that artemisinin significantly inhibited the growth of the wild-type *C. glabrata* strain [24]. Another study found that *C. glabrata, C. guilliermondii,* and *C. parapsilosis* were the most susceptible to artemisinin [19]. Consistent with these findings, our study showed that artemisinin was more effective against non-albicans species, including *C. krusei*, *C. tropicalis*, *C. guillermondii*, *C. kefyr*, and *C. lusitanae.*

Furthermore, artemisinin is significantly more efficacious than fluconazole against the intrinsically resistant *C. krusei* strain. To our knowledge, this effect of artemisinin has been demonstrated for the first time in the literature in *C. krusei*.

Growing resistance to existing antifungals in recent years has drawn attention to key virulence factors in *C. albicans*, particularly the yeast-to-hyphal transition and transcription factors. Consequently, a new antifungal drug development strategy has focused on antivirulence drugs that inhibit biofilm formation. A few studies have shown that artemisinin and its derivatives can inhibit hyphae development and disrupt the biofilm structure of *C. albicans* in a dose-dependent manner [18,19,25]. Moreover, Liang et al. showed that artemisinin reduced *Candida* damage to oral epithelial cells to confirm whether hyphal inhibition could suppress infection capacity [25]. Supporting previous studies, our findings showed a significant inhibition of biofilm formation in the presence of artemisinin at 640 µg/mL.

Yeast-to-hyphae transition is important for various processes such as biofilm formation and host cell invasion. The RAS1-cAMP-EFG1 pathway plays a central role in regulating the transition from yeast to hyphae in *C. albicans*. This pathway is activated by stimulation of the membrane protein RAS1. This stimulation induces adenylyl cyclase (CYR1) to produce the second messenger cyclic AMP and protein kinase A (PKA) to affect signal transduction [32,33]. EFG1, one of the main targets of PKA, is a critical transcription factor involved in the white/opaque transition and biofilm formation of *C. albicans*. EFG1 has been reported to decrease after hyphal induction; therefore, it is essential in the initial transition to hyphae [34]. EFG1 also regulates the expression of adhesins such as ALS, HWP1, and ECE1 and hyphal growth transcription factors such as UME6 and HGC1 [34,35]. Previous studies have reported that UME6 expression is sufficient to generate a nearly complete hyphae population, as well as attenuated virulence and defective hypha elongation during infection both in vitro and in vivo in UME6-silenced strains [35,36]. Furthermore, UME6 was shown to be sufficient to induce HGC1 even under non-filament-inducing conditions [35]. Hyphal G-cyclin 1 (HGC1) regulates mycelial growth and prevents cells from detaching from hyphae. ALS3 and HWP1 are adhesins involved in cell–cell and cell–surface interactions in hyphae [37]. The deletion of ALS3 restricts adherence to various matrices; therefore, ALS3-deficient strains can proliferate mycelia but cannot form biofilms. Thus, ALS3 is a crucial target for research into the efficacy of new antibiofilm agents [38]. HWP1 is a critical hypha-associated adhesin involved in intracellular signaling, hyphae development, and cell wall assembly. Mutant strains lacking HWP1, which is responsible for the initial stage of colonization, have been reported to be unable to bind to epithelial cells [39]. During epithelial invasion, ECE1 is one of the genes highly expressed by hyphae. In addition, ECE1 elevated up to 10,000-fold within minutes after the induction of filamentous growth [40]. Understanding the functions of these molecules is essential for studying the mechanisms of hyphal growth and developing strategies to target biofilm formation and host–cell interactions.

Pharmacological inhibition of these key pathways of *C. albicans* virulence and targeting their transcription factors and molecular interactions can be promising drug targets for antifungal development [41]. Based on these data, we investigated the influence of artemisinin on the expression of genes related to cell adhesion and yeast hyphal transformation, such as ALS3, HWP1, ECE1, and HGC1, as well as the RAS1-cAMP-EFG1 signaling pathway in *C. albicans* biofilms. Our findings revealed that artemisinin suppressed the RAS1-CYR1-EFG1 pathway and EFG1-regulated genes (ALS3, HWP1, ECE1, and HGC1) at a 640 µg/mL dose within 24 h in *Candida* biofilms. Furthermore, we found that artemisinin exhibited increased suppression of biofilm-related genes, particularly the key regulator EFG1, in *C. albicans* ATCC 10231 compared to fluconazole. However, artemisinin similarly suppressed these pathways to fluconazole in clinical strains. In accordance with our findings, a recent study has shown that arteether, an artemisinin derivative, suppresses the RAS1-cAMP-EFG1 pathway in *C. albicans* in transcriptome analysis, thereby inhibiting hyphal development [25]. Unlike this study, our study showed that artemisinin can suppress adhesion genes such as HWP1, ALS3, and ECE1, as well as biofilm-related genes such as UME6 and HGC1. These results are consistent also with the cytopathological results showing that artemisinin causes damage to hyphae and irregular aggregation in yeasts.

Molecular docking is a valuable tool for studying interactions between molecules, especially in the context of enzymes, proteins, and their substrates or inhibitors. Molecular docking allows researchers to predict and analyze how a small molecule, such as a drug or substrate, interacts with an enzyme and protein at the molecular level. This can reveal the mechanism of action, which is critical to understanding how a compound affects a biological system [42]. This knowledge is crucial in drug discovery and design as it helps researchers optimize compounds for greater binding affinity and selectivity to the target enzyme [43,44]. Moreover, these findings contribute to the broader understanding of fungal virulence and biofilm formation.

### Limitations

This study has some limitations. It should be pointed out that the limited number of genes implicated in biofilm and adhesion formation cannot reflect the antibiofilm efficacy of artemisinin. However, we tried to support our hypothesis about artemisinin by corroborating our PCR results with FESEM and a molecular docking study. Another limitation is the lack of in vivo application of artemisinin to confirm its antifungal and antibiofilm activity. This may be useful for developing more potent therapeutic strategies targeting biofilm formation pathways. Addressing these limitations and challenges will allow a more comprehensive evaluation of artemisinin’s potential role as an antifungal and antibiofilm compound.

## 4. Material and Methods

### 4.1. Candida Strains

This study included 43 clinical *Candida* strains obtained from the mycology collection of Selçuk University Faculty of Medicine Hospital between February 2019 and February 2020. In addition, three reference strains were also utilized: *C. albicans* ATCC 10231, *C. krusei* ATCC 6258, and *C. parapsilosis* ATCC 22019. To identify the strains at the species level, two methods were performed: the germ tube test and tests using the VITEK 2 Compact System (Biomérieux, Marcy-l’Étoile, France). Furthermore, the species was identified by multiplex PCR using universal and species-specific primers designed in previous studies [45,46]. DNA sequencing of the internal transcribed spacer (ITS) 1 and ITS 2 gene regions was used to confirm inconsistencies between VITEK and multiplex PCR results. The *Candida* isolates were kept in 15% (*v*/*v*) glycerol at −80 °C until they were needed for testing. Before testing, the strains were thawed and subcultured on Sabouraud dextrose agar (Neogen Corporation, Lansing, MI, USA) at a temperature of 35 °C for 48 h to promote growth. 

### 4.2. Antifungal Susceptibility Testing

The antifungal susceptibility of the strains was evaluated using a modified broth microdilution technique based on the Clinical and Laboratory Standards Institute’s (CLSI) M27-A3 method [47]. Briefly, serial dilutions of artemisinin and fluconazole were prepared in RPMI 1640 (Gibco, MT, USA)-MOPS (AppliChem, Darmstadt, Germany) medium and added to test wells with concentrations of artemisinin (ChemCruz, Dallas, TX, USA) and fluconazole (ChemCruz, USA) ranging from 5 µg/mL to 2560 µg/mL and 0.25 to 128 µg/mL, respectively. *Candida* strains were suspended in RPMI 1640-MOPS medium at a final density of 0.5–2.5 × ·10^3^ CFU/mL, and 100 µL of fungal inoculum was added to the test wells containing the diluted compounds. Control wells were prepared with 5% DMSO (Thermo Fisher Scientific, Waltham, MA, USA) in RPMI-1640 MOPS medium. The cultures were incubated at 35 °C for 24 h, followed by visual reading to determine the minimum inhibitory concentration (MIC). The MIC was calculated as the lowest concentration of the drugs that inhibit *Candida* growth by 50%. Antifungal activities were evaluated in triplicate and at least three separate experiments were conducted. 

### 4.3. In Vitro Biofilm Formation Assay

The biofilm-forming assay was performed according to previously described methods [48,49]. Twenty-eight C. albicans strains were used in this study. Briefly, 200 µL of fungal suspension (1 × 10^6^ cells/mL) was transferred to 96-well flat-bottomed plates and inoculated at 37 °C for 90 min. Following initial adhesion, the medium was aspirated, and non-adherent cells were washed three times with sterile 1X PBS (AppliChem, Germany). Subsequently, 200 µL of fresh RPMI 1640-MOPS medium was inserted into each well. The plate was further incubated at 37 °C for 24 h until biofilm formation. To assess biofilm formation, the supernatant was gently removed. The biofilm was rinsed two times with 200 µL of 1X PBS and dried at 60 °C for 30 min. Upon drying, 50 µL of 1% *w*/*v* crystal violet solution (1% *w*/*v*, Carlo Erba Reagents, Milan, Italy) was dispensed into each well and incubated for a further 15 min. The plates were washed twice with 1X PBS to remove unabsorbed crystal violet solution. Then, 150 µL of absolute ethanol was added to the wells to release the dye from the biofilm. After that, the absorbance of the microplates was measured by the microtiter plate reader Multiskan Sky, Thermo Fisher Scientific, USA) at 590 nm. Based on the classification proposed by Stepanovic et al., the isolates were divided into four levels of adhesion: non-adherent, weakly adherent, moderately adherent and strongly adherent. All experiments were performed in triplicate to ensure reliability [50].

### 4.4. Biofilm Formation and Treatment

To assess the impact of artemisinin on *C. albicans* biofilms, the biofilms were cultivated on 96-well polystyrene plates using a method outlined in prior research [48,49,51]. After 90 min, to allow for cell adhesion, the wells were washed with PBS to remove non-adherent cells. Subsequently, a fresh RPMI-1640 medium infused with varying concentrations of artemisinin (ranging from 5 to 2560 µg/mL) and fluconazole (from 0.25 to 128 µg/mL) was introduced to the adherent cells. The plates were then incubated at 37 °C for an additional 24 h. The metabolic activity within the biofilm was measured using the 3-[4,5-dimethylthiazol-2-yl]-2,5 diphenyl tetrazolium bromide (MTT) assay. The media were aspirated, and the biofilm was rinsed with 1X PBS. Then, 20 µL of MTT solution (5 mg/mL; Bio Basic, Toronto, ON, Canada) was added to the wells with the prewashed biofilm as well as to the control wells. The plates were incubated at 37 °C for 4 h in the dark. Following the incubation, the supernatants were discarded, and 100 µL of DMSO was added to each well, followed by a further 10 min incubation at 37 °C. The optical density was then measured at 570 nm using a MultiSkan Sky Microplate Reader (Thermo Fisher Scientific, USA). These procedures were all performed in triplicate to ensure experimental consistency.

### 4.5. Determination of the Biofilm and Hypha-Specific Gene Expressions by Real-Time Polymerase Chain Reaction

To investigate the effect of artemisinin on biofilm-related genes, the reference strain *C. albicans* ATCC 10231 and two strains identified as strongly adherent in the biofilm assay, 7A and 66A, were used. First, *C. albicans* was cultured in YPD medium at 35 °C for 18 h in a shaking incubator at 75 rpm. Cell suspensions were prepared in RPMI 1640 at a final concentration of 1 × 10^6^ CFU/mL in a 150 mm culture dish (SPL Life Sciences, Pocheon, Korea), as described previously [51]. Afterward, the cells were incubated at 37 °C for 24 h with biofilm-inhibitory concentrations (640 µg/mL artemisinin and 4 µg/mL fluconazole) as determined by the MTT assay. The untreated cells were considered as the control group. The medium was removed at the end of the experimental period, and the cells were collected using a cell scraper. Subsequently, 1.5 mL of cold PBS was added and centrifuged at 3000× *g* for 2 min at 4 °C. Following the manufacturer’s protocol, total RNAs were isolated from the supernatant using the YeaStar RNA Kit (Zymo Research, Orange, CA, USA).

Total RNA concentration and quality were measured and converted to cDNA using a cDNA synthesis kit (iScript cDNA synthesis kit, Bio-Rad, Hercules, CA, USA). To measure the expression levels of HWP1, CYR1, HGC1, EFG1, UME6, ECE1, RAS1, and ALS3 genes, a 2X SYBR Green Master Mix (Roche, Basel, Switzerland) was used according to the manufacturer’s instructions. The specific primers used for quantitative real-time PCR (qRT-PCR) analysis are listed in Table 3. The relative expression levels of the target genes were analyzed using a QuantStudio™ 3 Real-Time PCR System (Thermo Fisher Scientific, USA). The 2^−ΔΔ^Ct formula was used to determine the relative change in mRNA expression levels, with the expression of 18S rRNA serving as an internal standard for normalization. The relative expression of each target gene was presented as a fold change relative to its corresponding expression in untreated cells.

### 4.6. Molecular Docking Studies

Molecular docking was used to elucidate the molecular interaction between artemisinin and *C. albicans* genes. Eight molecular targets associated with antifungal biological activity in *C. albicans* were selected for the study. As the target receptors to which the artemisinin will be docked, the crystal structures of RAS1-cAMP-EFG1 genes (RAS1 (AF ID: P0CY32), CYR1 (AF ID: A0A1D8PR83), and EFG1 (AF ID: Q59X67)) and EFG1-regulated genes (ALS3 (AF ID: A0A2H9ZSK1), HWP1 (AF ID: P46593), ECE1 (AF ID: Q07730), UME6 (AF ID: Q59MD2) and HGC1 (AF ID: Q5ABE2)) were selected. The protein structures were obtained from the AlphaFold protein structure database (https://alphafold.ebi.ac.uk, accessed on: 12 February 2024). Docking analyses were performed using the Molegro Virtual Docker MVD 2019.7.0 software (Molegro ApS, Aarhus, Denmark) [52], together with the EADock DSS algorithm (http://www.swissdock.ch, accessed on: 12 February 2024). The initial three-dimensional configurations of the compounds under study were prepared using GaussView, while the Gaussian 09 software facilitated the identification of the lowest energy structures through geometry optimization [53]. The optimized structures were then saved in the *.pdb file format.

### 4.7. Molecular Dynamics Simulation Studies

The stability of artemisinin with selected *C. albicans* genes during MD simulations is confirmed by the RMSD (root means square deviation) plot. The binding behavior of artemisinin was further analyzed using the GROMACS 2020 software [54]. The chosen receptors were then subjected to a 100 ns MDS analysis. All of the complex systems were solved with TIP3P water molecules, and sufficient amounts of Na+ and Cl were added to the systems to achieve a salt concentration of 0.15 M. The CHARMM36 force field was applied in all of the simulations [55]. After minimization of the whole system, the temperature was slowly adjusted to 310 K, which is close to the physiological temperature of the human body (37 °C). Production runs of the MD simulation were then carried out for 100 ns without any structural constraints.

### 4.8. Field Emission Scanning Electron Microscopy

The impact of artemisinin on *Candida* biofilms was examined using field emission scanning electron microscopy (FESEM), following a methodology similar to a prior study, with slight adaptations [56]. To observe the inhibition of biofilm formation by artemisinin, *C. albicans* biofilms were formed on special plastic coverslips (SPL Life Sciences, Korea). Suspensions of the *C. albicans* ATCC 10231 strain were added to the plastic coverslips in 12-well culture plates and incubated at 37 °C for 90 min to ensure adhesion. After the adhesion phase, artemisinin (640 µg/mL) and fluconazole (4 µg/mL) were added, and the coverslips were incubated for 24 h. Biofilms were rinsed with PBS and then fixed in a solution containing 2% (*v*/*v*) glutaraldehyde (Tekkim, Bursa, Turkey) for 1 h at 25 °C. The biofilms were then dehydrated with a graded series of ethanol (at concentrations of 10, 25, 50, 75, 90, 100%). After dehydration, the samples were sputter-coated with a 4 nm iridium layer using a high-vacuum coating device (Leica EM ACE600, Wetzlar, Germany). The coated coverslips were imaged using a field emission scanning electron microscope (ZeissGemini500, Oberkochen, Germany) with an energy-dispersive X-ray spectroscopy (EDS) attachment to obtain high resolution and quality.

### 4.9. Statistical Analysis

The results are expressed as mean ± standard error of the mean (SEM). Statistical analysis was conducted using two-way analysis of variance (ANOVA) and Tukey’s multiple comparison test with the GraphPad Prism software (version 9.00; GraphPad Software, Inc., San Diego, CA, USA). The graphical abstract was generated using Biorender.com. Statistical significance was determined by *p* values less than 0.05.

## 5. Conclusions

In conclusion, our study suggests that artemisinin may be a potential fungistatic antifungal compound for *C. albicans*, which acts through multiple mechanisms during biofilm development rather than exhibiting fungicidal activity by inhibiting *C. albicans* growth. Indeed, artemisinin-treated biofilms showed a significant reduction in biomass and thickness compared to untreated biofilms in cytopathologic images. This visual evidence supports the antibiofilm activity of artemisinin. Furthermore, molecular analyses revealed the interaction of artemisinin with key proteins involved in biofilm formation, such as adhesins and EFG1. Although these specific molecular interactions provide further evidence for the potential efficacy of artemisinin in inhibiting biofilm development, comprehensive studies are required. Moreover, although artemisinin has shown promise in inhibiting fluconazole-resistant non-albicans strains, further research is needed to determine its efficacy against a broader range of clinical isolates. In summary, our results suggest that artemisinin is a promising antibiofilm candidate and contributes to understanding its multifaceted therapeutic value.

## Figures and Tables

**Table 1:** The antifungal activity of the artemisinin and FLC against *Candida* strains.

Antifungal Activity MIC Range (μg/mL)		
*Candida* Species (n)	Artemisinin	FLC
*C. albicans (28)*	320–1280	0.5–4
*C. krusei* (4)	5	32–64
*C. tropicalis (4)*	160–320	1–4
*C. kefyr (3)*	80	1–4
*C. lusitaniae* (2)	160–640	1–4
*C. guilliermondii* (2)	20	2
*C. albicans ATCC 10231*	640	0.5
*C. krusei ATCC 6258*	10	128
*C. tropicalis NRRLY-12968*	320	8

**Table 2:** Numerical values of the parameters obtained from the interactions of artemisinin with biofilm-related genes in *C. albicans*.

Genes	MolDock Score	Steric Interaction	H.Bond kcal/mol	H.Bond Residue Lenght	Artemisinin–Genes *	Total Energy kcal/mol	ΔG_bind_ kcal/mol
EFG1	−129.466	119.340	−8.838	Arg 231 (2.862 Å)	C=**O**–N**H**	−128.187	−9.02
				Val 222 (2.699 Å)	C-**O**-O–N**H**		
				Val 222 (2.628 Å)	C-O-**O**–N**H**		
				Val 230 (2.262 Å)	C-O-**O**–N**H**		
UME6	−126.667	−116.532	−7.845	Leu 815 (2.820 Å)	C=**O**–N**H**	−127.199	−8.79
				Leu 815 (2.820 Å)	C-**O**–N**H**		
				Thr 816 (3.221 Å)	C-**O**–N**H**		
				Thr 816 (3.331 Å)	C-O-**O**–N**H**		
HGC1	−122.299	−114.834	−7.118	Leu 296 (2.334 Å)	C=**O**–N**H**	−125.250	−8.67
				Lys 127 (2.331 Å)	C-**O**–N**H_3_**		
				Lys 127 (3.099 Å)	C-**O**–N**H_3_**		
				Lys 127 (2.334 Å)	C-O-**O**–N**H_3_**		
RAS1	−119.998	−111.367	−6.123	Leu 81 (2.462 Å)	C=**O**–N**H**	−122.980	−8.02
				Val 9 (2.262 Å)	C-**O**–N**H**		
				Val 8 (2.862 Å)	C-O-**O**–N**H**		
CYR1	−116.250	−109.577	−5.844	Arg 657 (2.822 Å)	C-O-**O**–N**H**	−120.190	−7.86
				Arg 679 (2.851 Å)	C-**O**-O–N**H_2_**		
				Arg 679 (2.334 Å)	C-O–N**H_2_**		
HWP1	−115.699	−108.834	−4.891	Cys 347 (2.875 Å)	C-**O**–N**H**	−118.384	−7.67
				Cys 347 (3.099 Å)	C-O-**O**–N**H**		
				Tyr 346 (2.226 Å)	C-**O**–N**H**		
ECE1	−114.577	−88.834	−3.867	Val 77 (2.569 Å)	C-**O**–N**H**	−113.823	−7.02
				Ile 178 (2.628 Å)	C-**O**–N**H**		
ALS3	−113.367	−79.834	−3.144	Lys 24 (2.367 Å)	C-**O**–N**H_3_**	−110.144	−6.86
				Glu 157 (2.331 Å)	C=**O**–N**H**		

* The underlined parts indicate bonds.

**Table 3:** List of qRT-PCR primers used in this study.

Genes	Forward Primer Sequence (5′→3′)	Reverse Primer Sequence (5′→3′)
HWP1	GCTCCTGCTCCTGAAATGAC	CTGGAGCAATTGGTGAGGTT
CYR1	CCAACAAACGACCAAAAGGT	TCTTGAACTGCCAGACGATG
HGC1	GCTTCCTGCACCTCATCAAT	AGCACGAGAACCAGCGATAC
EFG1	GCCTCGAGCACTTCCACTGT	TTTTTTCATCTTCCCACATGGTAGT
UME6	ACCACCACTACCACCACCAC	TATCCCCATTTCCAAGTCCA
ECE1	TTGCTAATGCCGTCGTCAGA	GAACGACCATCTCTCTTGGCAT
RAS1	TGGATGTTGTGTTATTGTTTGAGC	GTCTTGAATTGTTCATCTTCTCCCA
ALS3	TCGTCCTCATTACACCAACCA	TGAAGTTGCAGATGGGGCTT
18S rRNA	AGAAACGGCTACCACATCCA	AGCCCAAGGTTCAACTACGA

## Data Availability

The data presented in the study are available on request from the corresponding author.

## Supplementary Materials

The following supporting information can be downloaded at: https://www.mdpi.com/article/10.3390/antibiotics13040310/s1, Table S1: The MIC values of the artemisinin and FLC against *Candida albicans* strains.
