# Supplementary material for: Artemisinin May Disrupt Hyphae Formation by Suppressing Biofilm-Related Genes of Candida albicans: In Vitro and In Silico Approaches
Source: Antibiotics (Basel). 2024 Mar 28;13(4):310. doi: 10.3390/antibiotics13040310 (PMC11047306; doi:10.3390/antibiotics13040310)
Supplement: Supplementary file 1 [file antibiotics-13-00310-s001.zip › antibiotics-2934777-supplementary.pdf]

Table S1. The MIC values of the artemisinin and FLC against *Candida albicans* strains.

Strain Number	Identification	Artemisinin	FLC
1A	*C. albicans*	640	1
2A	*C. albicans*	640	2
4A	*C. albicans*	1280	2
6A	*C. albicans*	1280	1
7A	*C. albicans*	320	1
9A	*C. albicans*	1280	2
10A	*C. albicans*	640	2
11A	*C. albicans*	320	0.5
13A	*C. albicans*	1280	2
15A	*C. albicans*	1280	2
17A	*C. albicans*	1280	1
18A	*C. albicans*	640	1
19A	*C. albicans*	640	2
21A	*C. albicans*	1280	1
26A	*C. albicans*	1280	1
27A	*C. albicans*	1280	4
28A	*C. albicans*	1280	1
29A	*C. albicans*	1280	1
31A	*C. albicans*	1280	2
61A	*C. albicans*	320	0.5
66A	*C. albicans*	640	1
67A	*C. albicans*	640	2
74A	*C. albicans*	640	2
75A	*C. albicans*	1280	2
76A	*C. albicans*	640	4
80A	*C. albicans*	640	2
88A	*C. albicans*	1280	1
94A	*C. albicans*	1280	4
62A	*C. lusitanae*	640	1
65A	*C. lusitanae*	160	4
22A	*C. kefyr*	80	1
71A	*C. kefyr*	80	4
97A	*C. kefyr*	80	2
56A	*C. guillermondii*	20	2
58A	*C. guillermondii*	20	2
54A	*C.tropicalis*	160	4
48A	*C. tropicalis*	160	2
55A	*C. tropicalis*	320	2
42A	*C. tropicalis*	160	1
35A	*C. krusei*	5	32
81A	*C. krusei*	5	64
82A	*C. krusei*	5	64
83A	*C. krusei*	5	64
47A	*C. tropicalis NRRLY-12968*	320	8
78A	*C. krusei ATCC 6258*	10	128
102A	*C. albicans ATCC 10231*	640	0.5
